# Mono- and biallelic germline variants of DNA glycosylase genes in colon adenomatous polyposis families from two continents

**DOI:** 10.3389/fonc.2022.870863

**Published:** 2022-10-28

**Authors:** Alisa Petriina Olkinuora, Andrea Constanza Mayordomo, Anni Katariina Kauppinen, María Belén Cerliani, Mariana Coraglio, Ávila Karina Collia, Alejandro Gutiérrez, Karin Alvarez, Alessandra Cassana, Francisco Lopéz-Köstner, Federico Jauk, Hernán García-Rivello, Ari Ristimäki, Laura Koskenvuo, Anna Lepistö, Taina Tuulikki Nieminen, Carlos Alberto Vaccaro, Walter Hernán Pavicic, Päivi Peltomäki

**Affiliations:** ^1^ Department of Medical and Clinical Genetics, Medicum, University of Helsinki, Helsinki, Finland; ^2^ Programa de Cáncer Hereditario (Pro.Can.He.), Hospital Italiano de Buenos Aires, Ciudad Autónoma de Buenos Aires, Argentina; ^3^ Instituto Multidisciplinario de Biología Celular (IMBICE), Comisión de Investigaciones Científicas de la provincia de Buenos Aires (CICPBA)-Consejo Nacional de Investigaciones Científicas y Técnicas (CONICET)-Universidad Nacional de La Plata (UNLP), Buenos Aires, Argentina; ^4^ Unidad de Proctología, Hospital de Gastroenterología “Dr. Carlos Bonorino Udaondo”, Ciudad Autónoma, de Buenos Aires, Argentina; ^5^ Centro de cáncer, Clínica Universidad de Los Andes, Santiago, Chile; ^6^ Unidad de Coloproctología, Clínica Las Condes, Santiago de Chile, Chile; ^7^ Servicio de Anatomía Patológica, Hospital Italiano de Buenos Aires, Ciudad Autónoma de Buenos Aires, Argentina; ^8^ Applied Tumor Genomics Research Program, Research Programs Unit, University of Helsinki, Helsinki, Finland; ^9^ Department of Pathology, Helsingin ja Uudenmaan Sairaanhoitopiirin Laboratorio (HUSLAB), Helsinki University Hospital and University of Helsinki, Helsinki, Finland; ^10^ Department of Gastroenterological Surgery, Helsinki University Hospital and University of Helsinki, Helsinki, Finland; ^11^ Instituto de Medicina Traslacional e Ingeniería Biomédica (IMTIB), Hospital Italiano de Buenos Aires (HIBA)-Instituto Universitario Hospital Italiano de Buenos Aires (IUHI)-Consejo Nacional de Investigaciones Científicas y Técnicas (CONICET), Ciudad Autónoma de Buenos Aires, Argentina

**Keywords:** DNA glycosylase, *NEIL1*, *OGG1*, *NTHL1*, *MUTYH*, polyposis, germline variant, exome sequencing

## Abstract

Recently, biallelic germline variants of the DNA glycosylase genes *MUTYH* and *NTHL1* were linked to polyposis susceptibility. Significant fractions remain without a molecular explanation, warranting searches for underlying causes. We used exome sequencing to investigate clinically well-defined adenomatous polyposis cases and families from Finland (N=34), Chile (N=21), and Argentina (N=12), all with known susceptibility genes excluded. Nine index cases (13%) revealed germline variants with proven or possible pathogenicity in the DNA glycosylase genes, involving *NEIL1* (mono- or biallelic) in 3 cases, *MUTYH* (monoallelic) in 3 cases, *NTHL1* (biallelic) in 1 case, and *OGG1* (monoallelic) in 2 cases. *NTHL1* was affected with the well-established, pathogenic c.268C>T, p.(Gln90Ter) variant. A recurrent heterozygous *NEIL1* c.506G>A, p.(Gly169Asp) variant was observed in two families. In a Finnish family, the variant occurred *in trans* with a truncating *NEIL1* variant (c.821delT). In an Argentine family, the variant co-occurred with a genomic deletion of exons 2 – 11 of *PMS2*. Mutational signatures in tumor tissues complied with biological functions reported for *NEIL1*. Our results suggest that germline variants in DNA glycosylase genes may occur in a non-negligible proportion of unexplained colon polyposis cases and may predispose to tumor development.

## 1 Introduction

Familial Adenomatous Polyposis (FAP; OMIM#175100) is characterized by multiple adenomas in the colorectum and an increased risk of colorectal cancer (CRC). In FAP, more than 100 adenomas are typically present, whereas an attenuated form (AFAP) is characterized by 10 – 100 polyps and a generally milder disease (1). Profuse or attenuated adenomatous polyposis was first associated with germline *APC* (OMIM*611731) variants in families exhibiting autosomal dominant inheritance, but has since been linked to biallelic *MUTYH* variants (MAP for *MUTYH*-associated polyposis; OMIM#608456) in families with autosomal recessive inheritance (2). *MUTYH* encodes a DNA glycosylase that acts on oxidative DNA damage by removing adenine misincorporated opposite 8-oxoG (3).

Up to 11-25% of FAP cases arise *de novo* (4, 5), and a fifth of *de novo* adenomatous polyposis cases are attributable to *APC* mosaicism (6). As much as 20% of cases with clinical features of FAP show no pathogenic variants in *APC*. Moreover, 80% patients with attenuated polyposis are molecularly unexplained (7–9). Recently, new predisposition genes for adenomatous polyposis have been identified, including *POLE* and *POLD1* (PPAP for polymerase proofreading associated polyposis; OMIM# 615083 and # 612591, respectively) (10), *AXIN2* (11), and biallelic *MSH3* (OMIM# 617100) (12) and *MLH3* (13) variants. Moreover, biallelic *NTHL1* (OMIM# 616415) variants have been associated with polyposis and multi-organ cancer predisposition (14). Except for *AXIN2*, all these genes contribute to DNA fidelity, through proofreading DNA after replication (*POLE* and *POLD1*), mismatch repair (*MSH3* and *MLH3*), or base excision repair (*NTHL1*). Like *MUTYH*, *NTHL1* targets oxidative DNA damage; it encodes a DNA glycosylase that repairs pyrimidine-derived oxidation products (3).

Inspired by recent findings of novel polyposis and cancer predisposition genes, we embarked on a study to uncover new molecular factors for unexplained polyposis cases across multiple populations by exome-wide screening. Our efforts revealed several families harboring potentially pathogenic germline variants in DNA glycosylase genes, including mono- and biallelic alterations of *NEIL1* (OMIM *608844; Endonuclease VIII-Like 1).

## 2 Materials and methods

### 2.1 Patient cohorts

**Figure 1:**
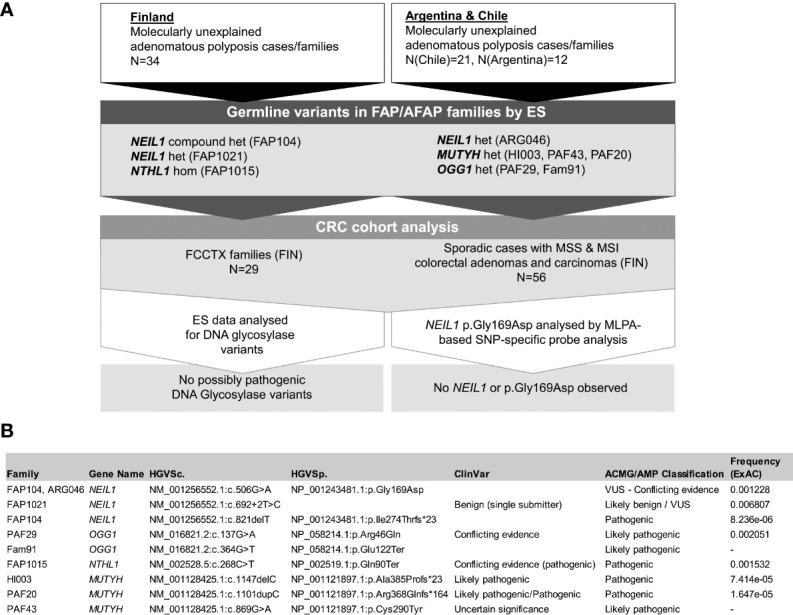
**(A)** Flowchart of the investigation including the Finnish and South American cohorts. Het denotes heterozygosity and hom homozygosity for the variants. **(B)** Detailed information of germline variants discovered in DNA glycosylase genes.

This investigation was based on 67 index cases with attenuated or profuse adenomatous polyposis (34 from Finland, 21 from Chile, and 12 from Argentina) in which known genetic causes of polyposis had been excluded (*APC*, *POLE*, *POLD1*, *PTEN* and biallelic *MUTYH*; **Figure 1**). The cases were ascertained through the national polyposis research registries and local hospitals as described below. Most cases (47/66, 71%) exhibited attenuated polyposis. Detailed clinical data are available in **Table S1**. Patient DNA was extracted from blood or EBV-transformed lymphoblasts as described by Renkonen et al. (15) DNA from formalin-fixed paraffin-embedded (FFPE) samples was extracted as described by Isola et al. (16) Patient RNA was extracted from lymphoblastoid cells using the NucleoSpin RNA extraction kit (Macherey-Nagel, Düren, Germany).

#### 2.1.1 Finnish cohort

Most polyposis cases were attenuated (22/33, 67%; one family could not be classified) and sporadic (20/28, 71%; six families could not be classified) (**Table S1**). An additional cohort of 29 families representing molecularly unexplained cases with familial colorectal type X (FCCTX) (17) and a series of sporadic cases (56 individuals) with microsatellite-unstable (MSI) CRC (N=13) or microsatellite-stable (MSS) CRC (N=44) or MSS adenomas (N=15) (18) were available for comparison (**Figure 1**).

#### 2.1.2 South American cohort

Thirty-three (21, Chile; 12, Argentina) unrelated families and index cases without known pathogenic variants in established colon polyposis-associated genes were screened as above. Most of the cases were attenuated (25/33, 76%) and had an apparent dominant mode of inheritance (16/28, 57%; five families could not be classified).

Written informed consent preceded study participation and sample donation. This study was approved by the institutional review board of the Helsinki University Central Hospital (Helsinki, Finland; Valvira/Dnro 10741/06.01.03.01/2015, 14.1.2016) and by the ethics committees of the Hospital de Gastroenterología “Dr. Carlos B. Udaondo” and Hospital Italiano de Buenos Aires (both from CABA, Argentina), and of the Clínica Las Condes (Santiago de Chile, Chile). The collection of archival specimens has been approved by the National Supervisory Authority for Welfare and Health (Valvira/Dnro 10741/06.01.03.01/2015, 14.1.2016).

### 2.2 Exome sequencing (ES) and germline variant selection

ES was performed at the Institute for Molecular Medicine Finland (FIMM, Helsinki, Finland) on Illumina HiSeq 2000 platform. The sequencing coverage and quality statistics for each sample are summarized in **Table S2**. Reads were aligned to the human reference genome hg19 using the Burrows-Wheeler Aligner version 0.6.2. Quality control and primary and secondary analysis were carried out as described by Sulonen et al. (19) Tertiary analysis was carried out using VarSeq^®^ software (Golden Helix). Variants with allele frequency <0.003, nonsynonymous (frameshift, stop gained/lost, missense, disrupting donor/acceptor site variants) and predicted pathogenic with at least five of six programs assessing protein function *in silico* (for missense changes) were selected. All variants in DNA glycosylase genes were confirmed by Sanger sequencing with primers listed in **Table S3**.

### 2.3 Copy number variant (CNV) analysis

CNV analysis on ES data was carried out using the R package ExomeDepth (v1.1.10) (20). The patient ES data was run against appropriate patient samples with known pathogenic changes using default settings and annotated using common CNV data from the DECIPHER database (https://www.deciphergenomics.org/). All samples had a correlation score >0.99. Only CNVs with a BF score of 10 or above were considered as candidate CNVs.

### 2.4 Characterization of *NEIL1* variants on DNA, RNA, and protein level

To confirm that the two coding variants detected in *NEIL1* in the index case of FAP104 affected different alleles (i.e., were *in trans*), cDNA was amplified with primers NEIL1_G83D_gcDNA_F and NEIL1_G83D_cDNA_R2 (**Table S3**) and cloned using the TOPO^®^ TA Cloning^®^ Kit for Subcloning (Thermo Fisher) according to manufacturer’s instructions. Transformed *E. coli* were then grown on selective plates (100 µg Ampicillin) overnight and white colonies were grown in LB (100 µg Ampicillin) overnight. Plasmids were extracted with GenElute™ Plasmid Miniprep Kit (Sigma-Aldrich) according to manufacturer’s instructions and Sanger sequenced using aforementioned primers as well as primers from Sjöblom et al. (21).

To evaluate allele-specific mRNA expression (ASE) in the lymphoblastoid cells from the index individual of FAP104, a Single Nucleotide Primer Extension (SNuPE) reaction was designed based on the heterozygous *NEIL1* c.506G>A variant. PCR products specific for cDNA (generated with primers NEIL1_G83D_gcDNA_F + NEIL1_G83D_cDNA_R2, **Table S3**) and gDNA (NEIL1_G83D_gcDNA_F + NEIL1_G83D_gDNA_R) served as templates for primer extensions with NEIL1_G83D_SNuPE_ext as the extension primer and ddA as the stopping nucleotide. The expected extension products were 34 bp (wild-type allele) and 24 bp (variant allele). Allele peak area ratios R<0.6 or R>1.67 indicated ASE (22).

*NEIL1* mRNA expression in the lymphoblastoid cells from the index individual of FAP104 and healthy controls was evaluated by quantitative reverse transcription PCR (qRT-PCR) with TaqMan^®^ Gene Expression Assay (Applied Biosystems) for *NEIL1* (Hs00908563_m1) and with glyceraldehyde-3-phosphate dehydrogenase (*GAPDH*) as an endogenous reference. The *NEIL1* reaction targeted exons 5 – 6 and covered the two main isoforms. The reactions were normalized against the *NEIL1* expression of healthy controls and the relative quantities were calculated using the ΔΔCT analysis.

To evaluate the stability of NEIL1 protein, lymphoblastoid cells from the index of FAP104 and unrelated healthy controls were treated with MG132 (Selleck Chemicals, Houston, Texas, USA). MG132 is a cell-permeable, proteasome inhibitor which reduces degradation of ubiquitin-conjugated proteins. Briefly, 0.5 x 10^6^ cells were incubated on 6 well plates for 8 hours and treated with 10, 30 or 50 µM MG132. Proteins from the cells were extracted in LAEMMLI extraction buffer.

NEIL1 protein expression in the treated and untreated lymphoblastoid cells from the index individual of FAP104 and healthy controls was assessed by Western blotting with the primary NEIL1 rabbit polyclonal antibody (12145-1-AP, RRID:AB_2251228; Proteintech, Rosemont, IL) targeting the NEIL1 short isoform (amino acids 1 – 390). The housekeeping protein glyceraldehyde-3-phosphate dehydrogenase (GAPDH) was used as a loading control (ab128915, RRID:AB_11143050; Abcam, Cambridge, UK). P53 (#9282 RRID:AB_331476; Cell Signaling Technology, Danvers, Massachusetts, USA) was used as a technical control for MG132 experiments.

### 2.5 *NEIL1* promoter methylation

A custom assay utilizing Methylation-Specific Multiplex Ligation-Dependent Probe Amplification (MS-MLPA) was designed to evaluate *NEIL1* promoter methylation in constitutional and tumor tissues. The *NEIL1* promoter region was investigated with four MS-MLPA probe pairs (**Table S3**), of which NEIL1_1 is located just upstream of the area found to be the most informative for methylation by Chaisaingmongkol et al. (23, 24).

### 2.6 Somatic variant profiling

VarScan2 variant detection algorithm version 2.3.2 was applied to tumor-normal pairs to identify non-synonymous somatic variants from ES data. Annotation of the variants was done using SnpEff version 4.0 with the Ensembl v68 annotation database (https://www.ensembl.org). Variants with a somatic p-value less than 0.01 were selected for somatic mutational signature analysis, which was carried out using the R package MutationalPatterns (25). The signatures were mapped against the 30 single-base substitution (SBS) and 18 insertion-and-deletion (ID) signatures recognized by the COSMIC database (v2 for SBS and v3.1 for ID, respectively, cancer.sanger.ac.uk).

### 2.7 Analyses for mismatch repair (MMR) and *MUTYH* status

A colorectal tumor from ARG046 was investigated for MMR protein expression by standard immunohistochemical procedures (26). Primary antibodies used were as follows (Roche Ventana, Indiana, USA): Anti-MLH1 (M1; 790-4535, RRID:AB_2336022), anti-MSH6 (44; 790-4455, RRID:AB_2336020), anti-MSH2 (G219-1129; 760-4265, RRID:AB_2336002), and anti-PMS2 (EPR3947; 760-4531, RRID:AB_2336010). DNA from the same tumor was evaluated for *MLH1* promoter methylation by MS-MLPA using the SALSA MLPA ME011-B3 probemix (MRC-Holland, Amsterdam, the Netherlands).

Blood DNAs from the index individuals from our polyposis cohorts were evaluated for large rearrangements in MMR genes and *MUTYH* by multiplex ligation-dependent probe amplification (MLPA) according to the manufacturer’s (MRC-Holland, Amsterdam, the Netherlands) instructions. SALSA MLPA P003-D1 and SALSA MLPA P072-D1 were used for *MLH1*/*MSH2* and *MSH6*/*MUTYH*, respectively, whereas *PMS2* was investigated by SALSA MLPA P008-C1. The results from fragment analysis were analyzed by Coffalyser™ (MRC-Holland, Amsterdam, the Netherlands).

### 2.8 Statistical analyses

Methylation ratios in sporadic tumors vs. matching normal tissues obtained from MS-MLPA analyses (**Table S4**) were compared using the Wilcoxon matched pairs test. IBM^®^ SPSS^®^ software (IBM SPSS Statistics 27, Armonk, NY: IBM Corp) was used for the analysis.

## 3 Results

We investigated the exomes of 67 index cases with molecularly unexplained polyposis from two continents, focusing on genes from the DNA glycosylase family. Pathogenic and likely pathogenic germline variants as well as VUSes whose pathogenicity is unknown but that have the potential of being pathogenic are described in **Figure 1** and **Table S5**. All germline variants fulfilling our selection criteria are listed for the DNA glycosylase-associated families in **Table S6**.

### 3.1 Germline DNA glycosylase variants found in the Finnish series

The European pathogenic founder variant *NTHL1* c.268C>T, p.(Gln90Ter) (14) was detected in a homozygous state in the index individual from FAP1015 (**Figure 1**). This individual had attenuated polyposis and was the only member with colorectal tumor manifestations in the family (**Table S1**). The patient was additionally diagnosed with carcinomas of multiple organs characteristic of the tumor spectrum of *NTHL1*-associated polyposis (27).

**Figure 2:**
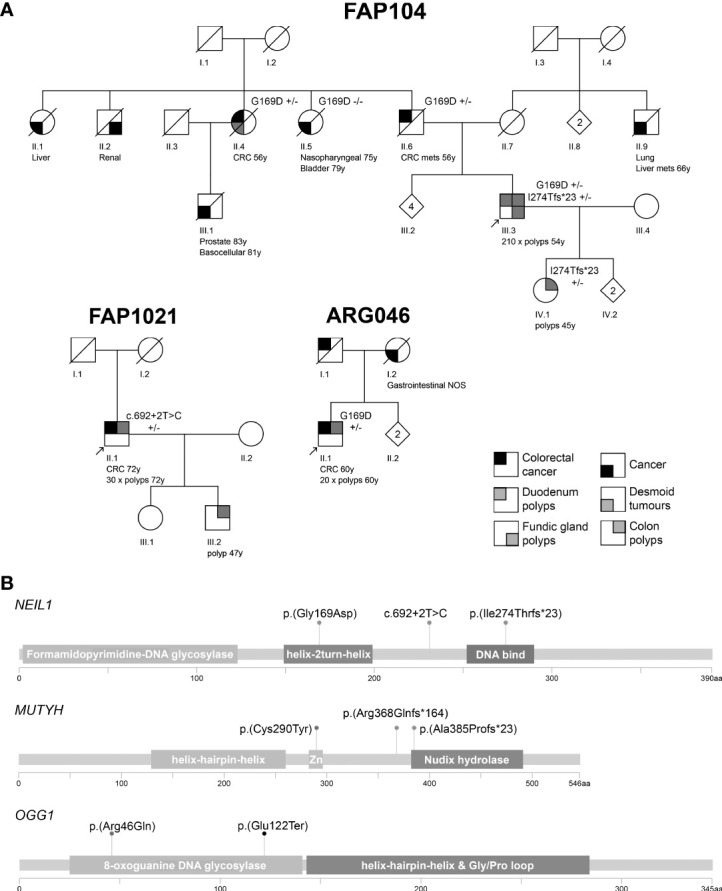
**(A)** Pedigrees of the polyposis families with *NEIL1* variants. Numbers below the symbols are patient identifiers. Arrow denotes the index person. Zygosity of *NEIL1* variants is shown (+/− heterozygous). Tumor manifestations and age at diagnosis (years) are given below the patient symbol. Mets refers to metastasis. Nonessential pedigree features were removed or modified to protect confidentiality. **(B)** Locations of the variants relative to the main functional domains of the DNA glycosylase genes. Zn denotes the metal binding sites in *MUTYH* as listed in the Uniprot database (www.uniprot.org; Q9UIF7). The pedigrees were generated with Pedigree Chart Designer and the lollipop diagrams with MutationMapper.

*NEIL1* variants were identified in FAP104 and FAP1021. The index of FAP104 with profuse polyposis (>200 polyps at 54 years of age) had two *NEIL1* variants (**Figure 1**, **Figure S1**); a rare missense variant c.506G>A, p.(Gly169Asp), and a very rare frameshift variant c.821delT, p.(Ile274Thrfs*23), absent in the Finnish population. A subsequent cloning assay revealed that the variants affected different alleles. All three individuals with the *NEIL1* c.506G>A variant had colorectal disease (cancer or polyps) and the same applied to the two individuals with the c.821delT variant (**Figure 2**).

**Figure 3:**
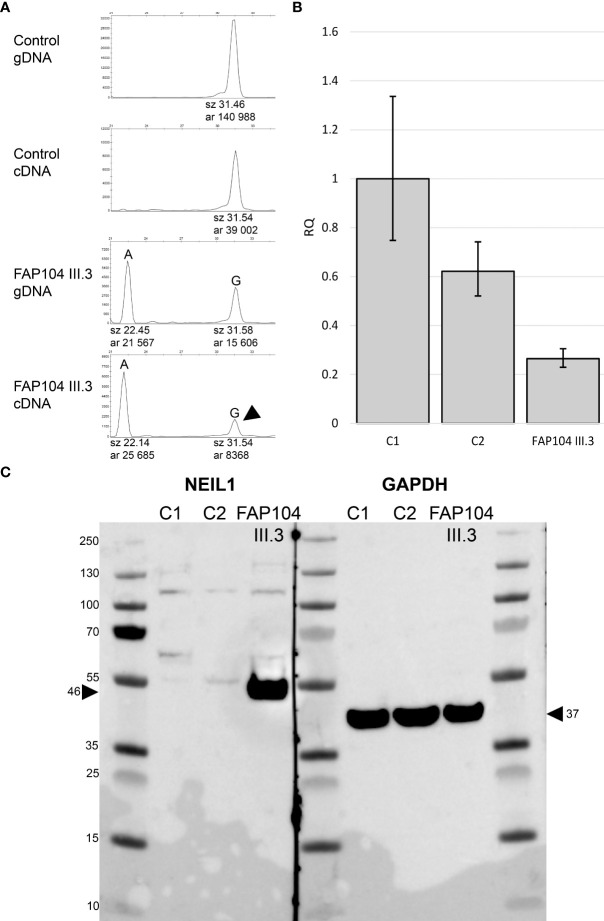
Expressional consequences of the *NEIL1* c.506G>A, p.(Gly169Asp) variant. **(A)** ASE analysis based on the *NEIL1* c.506G>A variant. Longer peak (G) represents the wild type sequence. The control individual is homozygous for the wild-type allele. The index individual of FAP104 is heterozygous: Allele A corresponds to the *NEIL1* c.506A missense variant, whereas the G allele is known to have a frameshift variant (c.821delT) in a downstream position. This individual displays ASE with the peak area ratio of 0.45 for G to A in cDNA relative to gDNA. The result indicates that transcripts with G (arrowhead) having the frameshift variant are twice less abundant than transcripts containing the missense variant **(A)**. **(B)** Relative quantity (RQ) from the qRT-PCR experiment targeting the two main isoforms of *NEIL1* using the housekeeping gene *GAPDH* as an endogenous control. Whiskers indicate 95% confidence limits. The index of family FAP104 shows reduced *NEIL1* RNA expression compared to the controls. **(C)** Western blot of two healthy control individuals and the index of FAP104. GAPDH was used as a loading control. FAP104 index displays elevated NEIL1 protein levels compared to the controls (arrowhead). No truncated NEIL1 protein is seen.

Conflicting evidence exists regarding the pathogenic significance of *NEIL1* c.506G>A (**Figure 1**, **Table S5**). The variant allele frequency in the (global) population (0.001228) is higher than expected for a dominantly inherited disorder when comparing against *NEIL1* variants reported through diagnostics. However, allele frequency of this variant in Finns (0.0001368) is almost ten times lower. Furthermore, previous functional studies conducted on *NEIL1* c.506G>A consistently suggest pathogenicity (see Discussion). *NEIL1* c.821delT is pathogenic according to the ACMG/AMP criteria (**Figure 1**, **Table S5**). Suitable biological specimens were available from the index of FAP104 to explore the consequences of the *NEIL1* variants on RNA and protein level. We evaluated the relative mRNA expression from the two *NEIL1* alleles by SNuPE and found that the frameshift variant containing transcripts were approximately twice less abundant than the missense variant containing transcripts in lymphoblastoid cells from the index individual of FAP104 (**Figure 3A**). By qRT-PCR, the total *NEIL1* mRNA expression was essentially lower than in healthy controls studied for comparison (**Figure 3B**), suggesting that the ASE seen by SNuPE was more likely to reflect decreased expression from the frameshift allele than increased expression from the missense allele. Interestingly, Western blot analysis revealed a markedly elevated amount of normalized full-length NEIL1 protein compared to healthy controls, and no truncated protein was visible (**Figure 3C**). The abundant full-length protein likely originated from the missense allele, and no stable protein was apparently generated from the frameshift variant containing allele. In the absence of increased *NEIL1* mRNA expression (**Figure 3B**), elevated NEIL1 protein in the Western blot was more likely to reflect aberrant protein stabilization than overexpression. The MG132 experiments (see Materials and Methods) did not reveal increased NEIL1 staining after treatment, indicating that regulation of NEIL1 protein expression is not MG132 mediated.

The index of FAP1021 with attenuated polyposis (30 polyps at 72 years of age) had a splice donor variant c.692+2T>C (**Figure 1**, **Figure S1**). In the literature, conflicting interpretations of pathogenicity for this splice variant exist (e.g., Dallosso AR et al. (28); Boldinova EO et al. (29)). In the absence of RNA, we were unable to experimentally verify splicing consequences of the variant. Based on available data, the ACMG/AMP classification is likely benign or VUS (**Table S5**). Available *in silico* software evaluated the splice donor variant highly likely to affect splicing (0.9918, 0.6039, 0.96, and 0.99683 for ADA, RF, SpliceAI, and SPiCE, respectively).

### 3.2 Germline DNA glycosylase variants found in the South American series

A patient from the Argentine family ARG046 with attenuated mixed polyposis and colorectal carcinoma at the age of 60 years was found to be heterozygous for the previously described *NEIL1* c.506G>A variant (**Figure 2**). No other possibly pathogenic variants in *NEIL1* were observed in the South American series.

Two families revealed likely deleterious *OGG1* variants. The index of family PAF29 with attenuated polyposis had a heterozygous missense variant of *OGG1*, c.137G>A, p.(Arg46Gln). In the literature, the same variant was described in a patient with synchronous colorectal cancer at 36 years and adenomas (30). It was shown that the G to A change which affects the last nucleotide of exon 1 disrupts a splice donor sequence, resulting in extinct expression from the variant allele in cDNA from the patient (30). The authors classified the *OGG1* c.137G>A variant pathogenic. Considering all available information, the ACMG/AMP criteria for likely pathogenic are fulfilled (**Figure 1**, **Table S5**). A heterozygous c.364G>T, p.(Glu122Ter) nonsense variant in the *OGG1* gene, likely pathogenic by the ACMG/AMP criteria (**Figure 1**, **Table S5**), was detected in family 91. The variant was present in the index patient (ID 606) with attenuated polyposis but absent in the index patient’s brother (ID 657) with late-onset colorectal carcinoma (**Table S1**).

Three heterozygous *MUTYH* variants were observed in the South American series (**Figure 1**, **Figure S2**). By ES, a frameshift variant of *MUTYH*, c.1101dupC, p.(Arg368Glnfs*164), was present in three individuals (ID 47, 534, and 535) out of four with colorectal adenomas or carcinoma from the Chilean family PAF20 and affected two generations. *MUTYH* c.1147delC, p.(Ala385Profs*23) was observed in the index individual HI003 (no other affected members were known to exist in this family). Both *MUTYH* variants described above are pathogenic by the ACMG/AMP criteria (**Table S5**), with biallelic involvement linked to MAP. A missense variant, *MUTYH* c.869G>A, p.(Cys290Tyr), classified as likely pathogenic (**Table S5**), was found in the index individual of family PAF43 (carrier statuses of the remaining family members were unknown). This family showed features of MAP (over 100 polyps in the index individual and an apparent recessive transmission pattern, **Figure S2**), raising the possibility that the *MUTYH* allele currently considered wildtype might harbor a defect that had escaped detection. However, manual IGV analysis of the gene and MLPA (with *MSH6-MUTYH* and *APC* MLPA kits) for large genomic rearrangements provided no support for biallelic *MUTYH* involvement.

### 3.3 Mutational analyses on tumors from individuals with *NEIL1* variants

DNA was available from a colorectal tubular adenoma from the index of FAP104 (compound heterozygous *NEIL1*; c.506G>A and c.821delT), two desmoid tumors from the paternal aunt of the index of FAP104 (heterozygous *NEIL1* c.506G>A), and a colorectal carcinoma from the index of ARG046 (heterozygous *NEIL1* c.506G>A) for somatic mutational profiling. We first determined the total mutational loads, since elevated numbers of somatic variants may point to defects in DNA replication or repair (10, 15). The total numbers of somatic nonsynonymous variants were 281 (adenoma), 45 and 57 (desmoids), and 1146 (carcinoma) by VarScan2 analysis. Based on the commonly used threshold of 10 variants/Mb, only the carcinoma of ARG046 was hypermutated (35 somatic variants/Mb).

All somatic variants meeting our selection criteria (VarScan2 p<0.01) are listed in **Table S7**. No somatic variant or loss of heterozygosity of *NEIL1* was observed in any sample. Thus, there was no evidence of a somatic “second hit” to the remaining wildtype allele in the monoallelic *NEIL1* variant carriers. The adenoma from the index of FAP104 showed a truncating *APC* variant (c.4666dupA, p.Thr1556fs; variant allele frequency (VAF) 23%) and *KRAS* c. 35G>C, p.Gly12Ala (VAF 25%), both representing alterations typical of colorectal tumorigenesis. The two desmoid tumors revealed extensive sharing of somatic variants, suggesting a common origin for the tumors.

**Figure 4:**
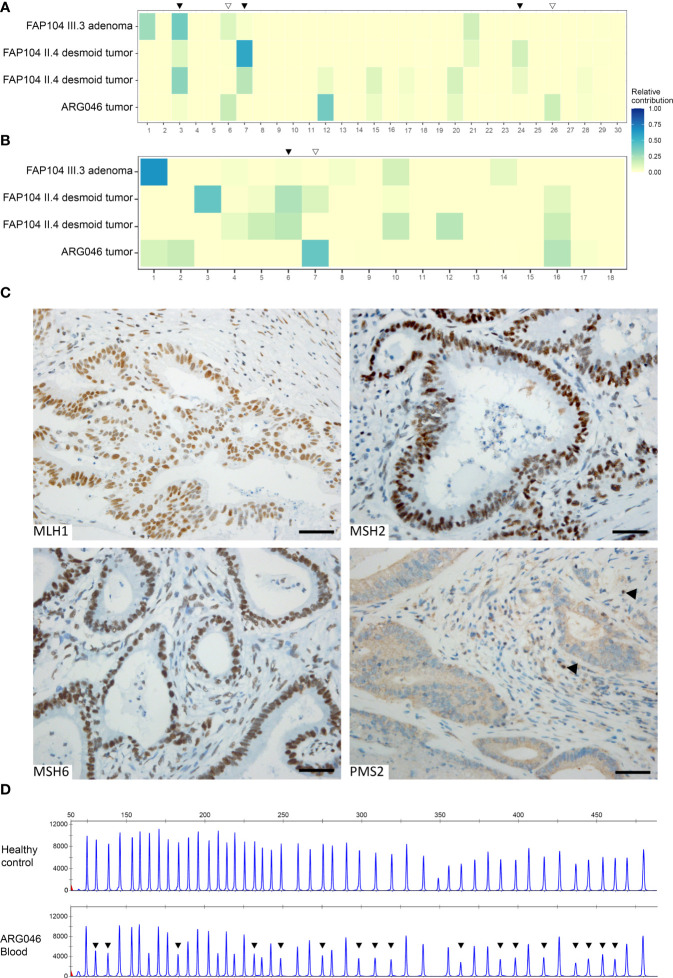
Somatic mutational signature analysis of four *NEIL1*-associated tumors **(A, B)**. **(A)** Heatmap indicating the relative contribution of SBS signatures (COSMICv2) to the mutational landscape of each tumor. Black arrowheads indicate *NEIL1-*deficiency associated signatures prominent in tumors from FAP104, whereas open arrowheads represent MSI-signatures present in a colorectal carcinoma from ARG046. **(B)** Heatmap of the relative contributions of ID signatures (COSMICv3.1, GRCh37) to the mutational profiles of the tumors. Subsequent discovery of the *PMS2* alteration in the ARG046 case **(C, D)**. **(C)** Immunohistochemical analysis of the MMR proteins reveals a selective loss of PMS2 in the tumor cells. Normal cells retaining the PMS2 expression are indicated with arrowheads. Scale bar represents 50 μm. **(D)** *PMS2*-MLPA analysis of blood DNA of the ARG046 case as well as a healthy control. Arrowheads indicate reduced emission peaks at exons 2-11. The average probe ratios of exons 2-11 (0.54 ± 0.03) are indicative of a heterozygous deletion.

As the patterns of somatic variants can offer insights to the underlying biological processes, a mutational signature analysis was conducted on the tumors (**Figures 4A, B**). VarScan2-based somatic variants were included in this analysis. COSMIC (31) SBS signature 3 (defective homologous recombination) was prominent in all three tumors from FAP104 (**Figure 4A**). Desmoid tumors from individual II.4 additionally revealed SBS7 (ultraviolet radiation exposure) and a discernible SBS24 linked to aflatoxin-associated mutagenesis (32). Interestingly, the hypermutable colorectal carcinoma from ARG046 showed prominent MMR deficiency-associated signatures SBS6 and SBS26, together with SBS12 (unknown etiology). The ID signature 6 supported defective homologous recombination in tumors from FAP104, whereas ID7 was compatible with deficient MMR in the colorectal carcinoma from ARG046 (**Figure 4B**).

### 3.4 *PMS2* genomic deletion found in the index case of ARG046

To resolve the MMR-deficient pattern of somatic alterations in the colorectal carcinoma from ARG046, the tumor was tested for *MLH1* promoter methylation, but no hypermethylation was present. However, immunohistochemical analysis revealed selective absence of PMS2 protein (**Figure 4C**). Subsequent MLPA analysis of blood DNA showed a heterozygous deletion of *PMS2* exons 2 - 11 (NM_000535.5:c.(23 + 1_24-1)-(2006 + 1_2007-1)del; **Figure 4D**). No additional cases with large rearrangements of MMR genes were detected when our entire polyposis series was evaluated by MLPA (and no small sequence alterations with possible pathogenicity existed in MMR genes by ES).

### 3.5 Methylation status of *NEIL1* in the polyposis and control cohorts

As *NEIL1* is commonly hypermethylated in cancer (23, 24), we designed a MS-MLPA kit to determine constitutional and somatic methylation status of our patient samples. Of the four MS-MLPA probe pairs, NEIL1_1 interrogated a region previously shown to be informative for methylation (23, 24) and showed the best discrimination between normal and tumor tissues (**Table S4**). Blood and normal colonic mucosae even from reference individuals revealed considerable methylation, and examination of blood DNAs from our polyposis cases (with or without *NEIL1* variants) raised no suspicion of constitutional *NEIL1* epimutation in any case. Compared to paired normal tissues, tumors from individuals with *NEIL1* variants occasionally displayed higher methylation dosage ratios, but no significant somatic hypermethylation of the promoter region was evident. Comparing paired tumor and normal tissues from sporadic cases with MSS or MSI carcinomas or adenomas revealed no significant difference by Wilcoxon matched pairs test (*Z*=-1.03, *p*=0.133 for MSI carcinomas vs matching normal tissues, and *Z*=-0.217, *p*=0.828 for MSS carcinomas vs matching normal tissues by *NEIL1* I probe, respectively).

## 4 Discussion

The DNA glycosylase family comprises eleven members, of which some (e.g., *MUTYH*) are monofunctional (capable of excising damaged or mispaired bases) and some (e.g., *NTHL1*, *OGG1*, and *NEIL1*) bifunctional (additionally having endonuclease activity to incise the modified strand) (33). The role of DNA glycosylases other than *MUTYH* and *NTHL1* in (colon) tumor susceptibility is unknown and/or associated with conflicting evidence, which encouraged us to undertake the present study. In our exomic screen of 67 index cases from Finnish and South American cohorts, 9 (13%) revealed proven or potentially pathogenic germline variants affecting *NEIL1* (3 cases), *MUTYH* (3 cases), *NTHL1* (1 case), and *OGG1* (2 cases). The findings suggest that germline variants in DNA glycosylase genes may explain a nontrivial proportion of unexplained cases of colorectal polyposis.

In our investigation, *NTHL1* showed biallelic involvement, consistent with the recessive *NTHL1*-associated polyposis syndrome (14). Our *OGG1* variants were monoallelic and suggested dominant transmission with reduced penetrance, which agrees with available literature (30), although a single case with a biallelic truncating *OGG1* variant was recently reported in association with FCCTX (34). Biallelic germline variants of *MUTYH* underlying the well-defined recessive MAP syndrome (2) were excluded from our series at the outset; the significance of the observed monoallelic *MUTYH* variants will be addressed below. Finally, the transmission pattern of *NEIL1*-associated disease is unclear since no segregation studies for *NEIL1* variants have been reported before. We detected one biallelic and two monoallelic *NEIL1* cases that will be discussed in more detail below.

The *NEIL1* missense variant c.506G>A, p.(Gly169Asp) occurred in two polyposis families, Finnish and Argentine (2/67, 3%). This variant was previously referred to as G83D according to annotation based on the short (390 amino acid) isoform. Forsbring et al. (35) found this variant in two patients among 37 with primary sclerosing cholangitis and cholangiocarcinoma (5%). Biochemical studies have provided consistent evidence that the variant is deleterious. Using 8-oxoG, thymine glycol, and 5-OHU as substrates, the NEIL1 Gly169Asp protein was found to be devoid of DNA glycosylase activity (35–38). Galick et al. (38) additionally showed that the variant NEIL1 protein acted as a dominant negative manner relative to the wild-type protein, being able to bind to damaged DNA but unable to repair it. Roy et al. (37) concluded that in individuals with the Gly169Asp variant, NEIL1 function is likely to be 50% compared to normal levels unless compensatory mechanisms exist. Our Western blot analysis on lymphoblastoid cells from the index of FAP104 with the c.506G>A variant revealed strikingly increased amount of NEIL1 protein, and we hypothesize that the c.506G>A variant is mutagenic due to the accumulation of functionally defective protein. Our result would comply with a possible oncogenic role proposed for *NEIL1* in some studies (39).

The index individual of FAP104 was compound heterozygous for *NEIL1* c.506G>A and c.821delT. We are not aware of the possible existence of any previous reports of biallelic constitutional *NEIL1* involvement in association with human disease. Moreover, in FAP104, all five members who were verified to have either one of the *NEIL1* variants (or both) had a colorectal tumor phenotype. The age at onset of disease (polyposis or cancer) of our heterozygous cases was relatively late with modest numbers of polyps (**Figure 2**, **Table S1**), which may indicate reduced penetrance. Apart from colon polyposis, profuse gastric fundic gland polyposis was apparent in the index of FAP104 as well as his daughter, both individuals with the *NEIL1* frameshift variant (**Figure 2**). Stomach tissue is particularly prone to oxidative damage and some somatic *NEIL1* variants and germline polymorphisms have been found in gastric cancer patients (40) indicating a possible role in stomach polyp formation.

SBS3 and ID6, which are associated with impaired homologous recombination (41), stood out among mutational signatures observed in our *NEIL1*-associated tumors (**Figures 4A, B**). This is compatible with observations that NEIL1 may participate in the repair of oxidized bases in D-loops (42) and R-loops (43) arising during homologous recombination or transcription. SBS7 which is connected to UV radiation was prominent in the desmoid tumors from a case with the *NEIL1* c.506G>A variant. This is consistent with findings of *Neil1^-/-^* mice being sensitive to chronic UVB exposure (44). Our desmoid tumors also exhibited SBS24, the so-called aflatoxin signature. McCullough and Lloyd (32) demonstrated that *NEIL1* is a major contributing factor to the repair of AFB_1_-N7-dG and AFB_1_-Fapy-dG adducts formed by aflatoxin mutagenesis. All in all, mutational signatures observed in tumors from our *NEIL1* cases are well in agreement with the reported biological consequences of defective *NEIL1* function.

Somatic hypermutability and MMR deficiency-associated signatures in a colorectal tumor from an Argentine case with the *NEIL1* c.506G>A variant provided critical clues to discover a large genomic deletion of *PMS2* as a concomitant germline alteration in this patient (**Figures 4C, D**). In analogy to DNA glycosylase genes, incomplete penetrance characterizes many pathogenic variants of *PMS2* (45). Colonic polyposis commonly accompanies biallelic *PMS2* variants, whereas monoallelic *PMS2* variants typically manifest themselves as (late-onset) colorectal carcinoma (46). The *PMS2* exon 2 – 11 deletion found in our ARG046 case was heterozygous (**Figure 3D**), and no other *PMS2* sequence variants of suspected pathogenic significance were identified (**Table S6**). Modifying or additive effects of two or more defective genes may be necessary to explain the observed phenotypes of DNA glycosylase gene variants (30) and *PMS2* variants (46), and base excision repair and MMR defects can potentiate each other’s effects (47). It is possible that the late-onset colorectal carcinoma in our ARG046 case mainly reflected the *PMS2* defect, in agreement with available literature (see above), whereas *NEIL1* c.506G>A might be necessary for the patient’s polyposis phenotype.

While biallelic germline variants of *MUTYH* cause predisposition to MAP (see Introduction), the clinical phenotype of monoallelic *MUTYH* variants remains unsettled. In our investigation, three families from the South American cohort revealed monoallelic *MUTYH* variants classified as pathogenic (two) and likely pathogenic (one). Among five individuals with monoallelic *MUTYH* variants, four exhibited polyposis with the polyp number ranging from below 20 (in three individuals) to over 100 (in one), and three had late-onset colorectal cancer (**Table S1**, **Figure S2**). Our findings together with published reports indicate that individuals with monoallelic *MUTYH* variants may be predisposed to colorectal polyposis of a variable degree and have a moderately increased risk of colorectal cancer (48, 49). The PAF43 index case manifested a phenotype akin to classical MAP, but no second *MUTYH* variant of possible pathogenic significance was identified. Since the ES runs included only about 300 bp flanking sequence, our approach does not exclude possible variants in regulatory regions (including deep intronic splice variants and pseudoexons).

In summary, we describe proven or possibly pathogenic germline variants of DNA glycosylase genes in 9/67 (13%) index cases with colon polyposis. Our study suggests a link between *NEIL1* germline variants and colon polyposis. Because of the relatively limited number of individuals with *NEIL1* variants in this investigation, our findings need to be confirmed in larger multinational cohorts.

## Data availability statement

The data analyzed in this study was obtained as described in Materials and Methods. The datasets are not readily available because our IRB approvals do not allow sharing raw exome sequencing data. However, all variants that fulfilled our selection criteria can be found in the **Supplementary Tables**. Requests to access these datasets should be directed to the corresponding author, APO, alisa.olkinuora@helsinki.fi.

## Ethics statement

The studies involving human participants were reviewed and approved by Helsinki University Central Hospital (Helsinki, Finland; Valvira/Dnro 10741/06.01.03.01/2015, 14.1.2016) Hospital de Gastroenterología “Dr. Carlos B. Udaondo” and Hospital Italiano de Buenos Aires (both from CABA, Argentina) Clínica Las Condes (Santiago de Chile, Chile) by the National Supervisory Authority for Welfare and Health (Valvira/Dnro 10741/06.01.03.01/2015, 14.1.2016). The patients/participants provided their written informed consent to participate in this study.

## Author contributions

Conceptualization, APO, PP, CAV, and WHP. Data curation, FIMM. Formal analysis, APO, AKK, PP, ACM, and MBC. Funding acquisition, PP, CAV, and WHP. Investigation, APO, AKK, AR, PP, ACM, MBC, FJ, HG-R, and WHP. Methodology, APO, AKK, TTN, PP, ACM, MBC, and WHP. Project administration, PP and WHP. Resources, LK, AL, PP, ACM, MBC, MC, AKC, AG, KA, AC, FL-K, CAV, and WHP. Software, APO. Supervision, TTN, PP, and WHP. Validation, APO, AKK, TTN, PP, ACM, MBC, and WHP. Visualization, APO, PP, and WHP. Writing-original draft, APO, PP, and WHP. Writing-review and editing, APO, PP, and WHP. All authors contributed to the article and approved the submitted version.

## Funding

This work was supported by grants from Biomedicum Helsinki Foundation (to APO); the Mary and Georg Ehrnrooth’s Foundation (to LK); the Jalmari and Rauha Ahokas Foundation (to TTN); the Maud Kuistila Memorial Foundation (to TTN); the Jane and Aatos Erkko Foundation (to PP); the Academy of Finland (grant number 330606 to PP); Cancer Foundation Finland sr (to PP, AR and LK); and Helsinki University Central Hospital Research Funds (to AR); the Sigrid Juselius Foundation (to PP and AR); the HiLIFE Fellows 2017-2020 (to PP); the Agencia Nacional de Promoción Científica y Tecnológica (PICT_2017 -3210 to CAV and -3197 to WHP) and Instituto Nacional del Cáncer Argentina (AF_IV to CAV and WHP). The Doctoral Programme in Biomedicine of Helsinki University offered a paid doctoral student position to APO.

## Acknowledgments

Saila Saarinen is thanked for expert laboratory assistance, and Maija Röntynen and Beatriz Alcala-Repo for collecting clinical data.

## Conflict of interest

The authors declare that the research was conducted in the absence of any commercial or financial relationships that could be construed as a potential conflict of interest.

## Publisher’s note

All claims expressed in this article are solely those of the authors and do not necessarily represent those of their affiliated organizations, or those of the publisher, the editors and the reviewers. Any product that may be evaluated in this article, or claim that may be made by its manufacturer, is not guaranteed or endorsed by the publisher.
