# Supplementary material for: Mono- and biallelic germline variants of DNA glycosylase genes in colon adenomatous polyposis families from two continents
Source: Front Oncol. 2022 Oct 28;12:870863. doi: 10.3389/fonc.2022.870863 (PMC9650540; doi:10.3389/fonc.2022.870863)
Supplement: Supplementary file 8 [file DataSheet_1.docx]

**Olkinuora et al. Supplementary files**

**
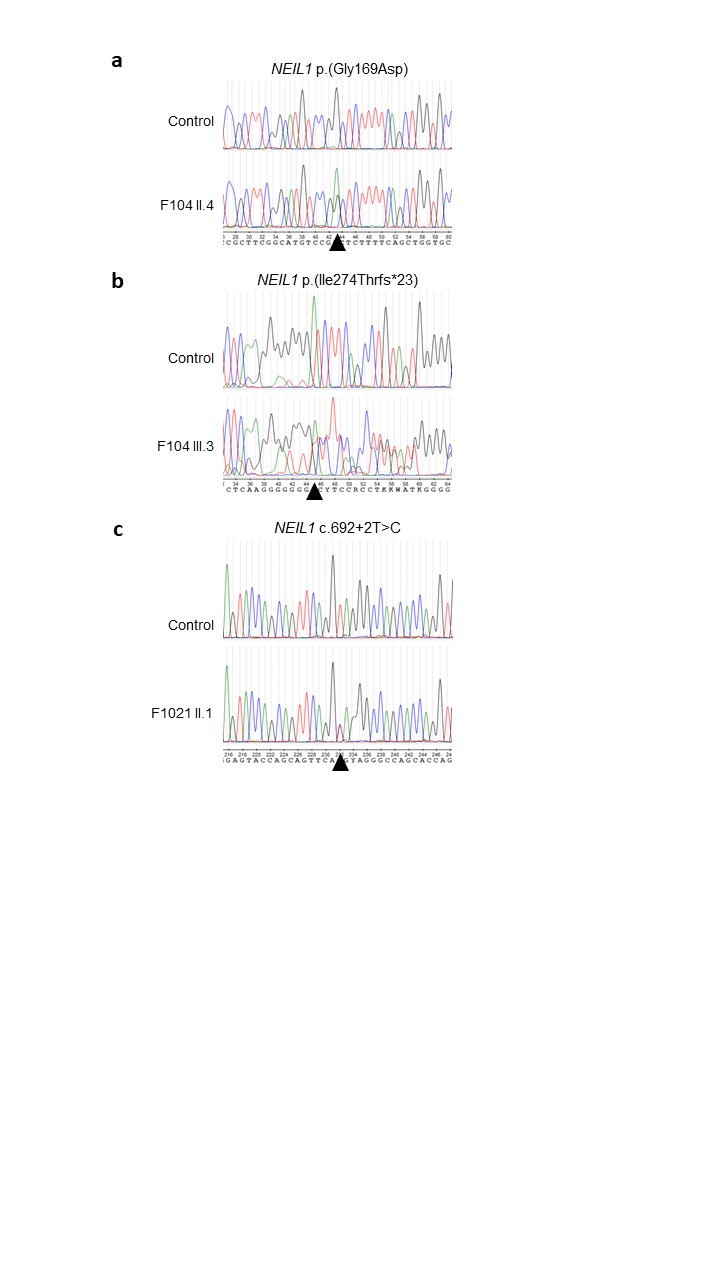
**

**Supplementary Figure 1. Sanger verification of *NEIL1* variants.** (a) *NEIL1* p.(Gly169Asp), (b) *NEIL1* p.(Ile274Thrfs*23), (c) *NEIL1* c.692+2T>C.

**
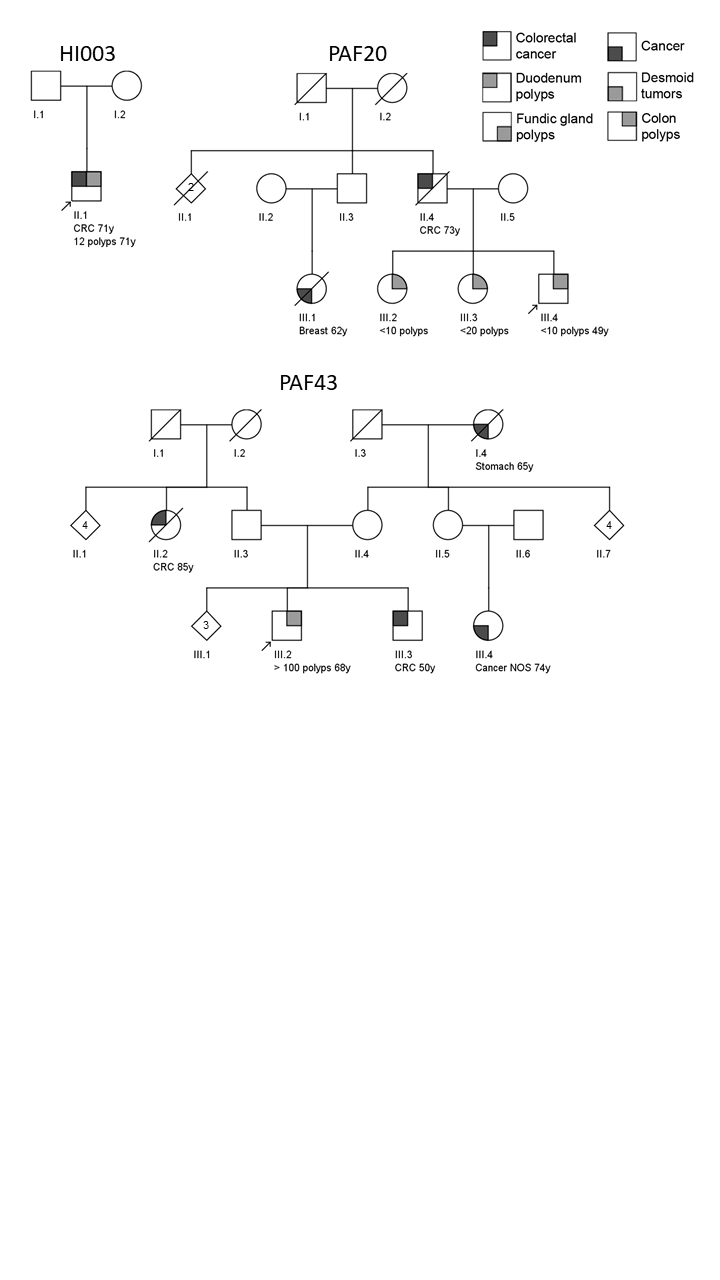
**

**Supplementary Figure 2. Pedigrees of the heterozygous MUTYH germline variant carriers.**
